# Recombinant ecto-5'-nucleotidase (CD73) has long lasting antinociceptive effects that are dependent on adenosine A_1 _receptor activation

**DOI:** 10.1186/1744-8069-6-20

**Published:** 2010-04-14

**Authors:** Nathaniel A Sowa, Meagen K Voss, Mark J Zylka

**Affiliations:** 1 Department of Cell and Molecular Physiology, UNC Neuroscience Center, University of North Carolina, CB #7545, Chapel Hill, North Carolina 27599, USA

## Abstract

**Background:**

Ecto-5'-nucleotidase (NT5E, also known as CD73) hydrolyzes extracellular adenosine 5'-monophosphate (AMP) to adenosine in nociceptive circuits. Since adenosine has antinociceptive effects in rodents and humans, we hypothesized that NT5E, an enzyme that generates adenosine, might also have antinociceptive effects *in vivo*.

**Results:**

To test this hypothesis, we purified a soluble version of mouse NT5E (mNT5E) using the baculovirus expression system. Recombinant mNT5E hydrolyzed AMP in biochemical assays and was inhibited by α,β-methylene-adenosine 5'-diphosphate (α,β-me-ADP; IC_50 _= 0.43 μM), a selective inhibitor of NT5E. mNT5E exhibited a dose-dependent thermal antinociceptive effect that lasted for two days when injected intrathecally in wild-type mice. In addition, mNT5E had thermal antihyperalgesic and mechanical antiallodynic effects that lasted for two days in the complete Freund's adjuvant (CFA) model of inflammatory pain and the spared nerve injury (SNI) model of neuropathic pain. In contrast, mNT5E had no antinociceptive effects when injected intrathecally into adenosine A_1 _receptor (*A*_1_*R, Adora1*) knockout mice.

**Conclusion:**

Our data indicate that the long lasting antinociceptive effects of mNT5E are due to hydrolysis of AMP followed by activation of A_1_R. Moreover, our data suggest recombinant NT5E could be used to treat chronic pain and to study many other physiological processes that are regulated by NT5E.

## Background

Ecto-5'-nucleotidase (NT5E) is a glycosyl phosphatidylinositol (GPI)-anchored membrane protein that catalyzes the hydrolysis of extracellular AMP to adenosine [1]. NT5E regulates diverse physiological processes that are modulated by adenosine, including hypoxia, inflammation and epithelial ion transport [2-8]. Recently, we found that NT5E is expressed in peptidergic and nonpeptidergic nociceptive (pain-sensing) neurons and their axon terminals in spinal cord and skin [9]. Based on experiments with *Nt5e*^-/- ^mice, we established that NT5E accounts for ~50% of all AMP hydrolytic activity in nociceptive neurons [9].

In addition, we observed that NT5E was extensively co-localized with Prostatic acid phosphatase (PAP, also known as ACPP, Fluoride-resistant acid phosphatase or thiamine monophosphatase) in nociceptive neurons. Like NT5E, PAP functions as an ectonucleotidase in nociceptive neurons by hydrolyzing AMP to adenosine [10,11]. Both *Pap*^-/- ^and *Nt5e*^-/- ^mice show enhanced thermal hyperalgesia in animal models of inflammatory pain and neuropathic pain as well as enhanced mechanical allodynia following inflammation [9,10]. Interestingly, *A*_1_*R*^-/- ^mice also show enhanced sensitization following inflammation and nerve injury [12]. Thus, deficiencies in adenosine production or A_1_R signaling cause similar behavioral phenotypes.

In support of an A_1_R-dependent mechanism, we found that intrathecal (i.t.) injection of secretory PAP protein (from mouse or human) into wild-type mice had long lasting antinociceptive, antihyperalgesic and antiallodynic effects that were entirely dependent on A_1_R activation [10,11]. These data suggested that spinal delivery of PAP protein could be used therapeutically to generate adenosine and activate A_1_R over an extended time period. Likewise, direct activation of A_1_R with adenosine or selective A_1_R agonists had antinociceptive effects in rodents and humans [13-31].

Considering that both PAP and NT5E generate adenosine, we hypothesized that NT5E protein might also have A_1_R-dependent antinociceptive effects. However, we were unable to test this hypothesis because mammalian NT5E protein was not available. As emphasized in a recent review by Colgan and colleagues, this lack of a reliable source of purified protein has hindered studies with NT5E [2]. Others used 5'-nucleotidase protein purified from rattlesnake (*Crotalus atrox*) venom instead [4,32,33]. *Crotalus *5'-nucleotidase had no deleterious effects when injected intraperitoneally and rescued phenotypes in *Nt5e*^-/- ^mice; however, possible toxicity from venom contaminants remains a concern--especially if this venom-derived protein were to be used in the nervous system.

To overcome these toxicity concerns, we purified and characterized (*in vitro *and *in vivo*) a secretory version of recombinant mouse NT5E. Our present study builds upon work by Servos and colleagues who purified a recombinant but non-secretory version of rat NT5E using the baculovirus expression system [34].

## Results

### Purification of soluble mouse NT5E using the baculovirus expression system

**Figure 1:**
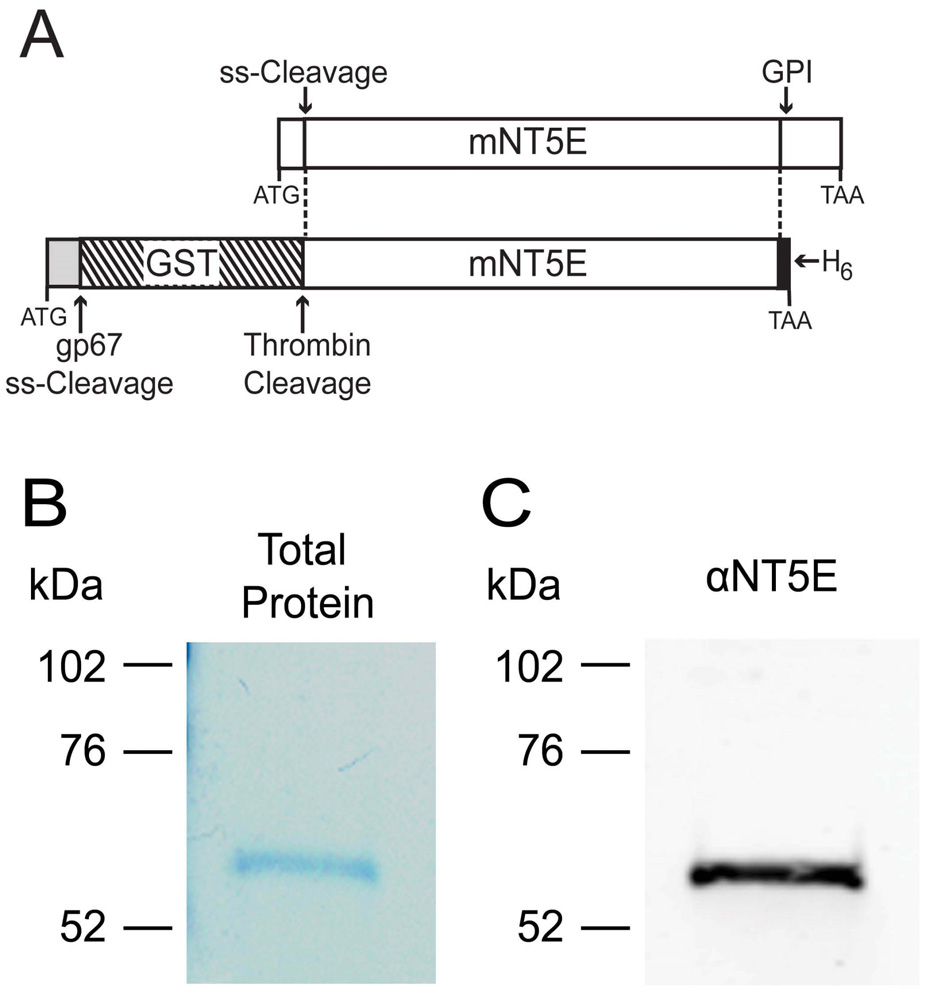
**Purification of recombinant mNT5E**. (A) Diagram of the GST-mNT5E expression construct. (Top) Native mNT5E contains an N-terminal signal peptide (ss-cleavage) and GPI anchor site. (Bottom) The GST-mNT5E fusion construct contains the signal peptide from gp67 of baculovirus *Autographica californica*, GST, a thrombin cleavage site, the catalytic domain of mNT5E and (His)_6 _tag. Translation start and stop codons are indicated. (B) GelCode blue-stained SDS-PAGE gel and (C) western blot of purified recombinant mNT5E protein (0.05 μg). The western blot was probed with an anti-mNT5E antibody.

NT5E is anchored to the membrane via a GPI linkage on Ser523 [35]. In an effort to produce a secreted and soluble (non-membrane anchored) version of NT5E, Servos and colleagues generated a baculovirus expression construct of rat NT5E that reportedly lacked the GPI-anchor at Ser523 [34]. While Servos and colleagues successfully used their construct to purify a catalytically active version of rat NT5E, they did not detect NT5E in the tissue culture medium as would be expected if the protein were secreted and soluble. Instead, NT5E was only present in cell lysates. Upon re-examination of the cloning strategy used by Servos and colleagues we noticed that their expression construct included Ser523 but excluded neighboring Ser526. Using GPI prediction software [36], we confirmed that Ser523 (but not Ser526) was the most likely GPI anchor site. Inclusion of this GPI anchor sequence could explain why Servos and colleagues did not detect NT5E in the culture medium. We thus generated a mouse NT5E (Trp29-Phe522) expression construct that was truncated just before Ser523 (Figure 1A). Our construct was otherwise identical to the one used by Servos and colleagues--our construct contained a gp67 signal peptide, glutathione S-transferase (GST), a thrombin cleavage site to permit removal of GST and a C-terminal hexahistidine (His)_6 _tag (Figure 1A).

Two days after infecting Hi5 insect cells with recombinant baculovirus we detected GST-mNT5E protein in the tissue culture supernatant at approximately 10 mg/L. This observation suggested that exclusion of Ser523 was important for producing a soluble version of NT5E. Additionally, based on SDS-PAGE and western blotting, the GST-mNT5E found in the medium was largely intact whereas cell lysates contained truncated and intact versions of mNT5E-GST (data not shown). We next purified mNT5E from the culture supernatant in two steps (see Methods). We reasoned that GST, a protein that binds glutathione, might interfere with physiological or behavioral studies if administered *in vivo*. So as part of our purification procedure, the GST fusion was removed by thrombin cleavage. Protein purity was analyzed under denaturing conditions with GelCode Blue protein stain (Figure 1B) and western blotting with an anti-NT5E antibody (Figure 1C). We observed a single band at ~62 kDa, corresponding to the calculated molecular weight of unglycosylated mNT5E with a (His)_6 _tag (61.7 kDa). No additional bands were observed, indicating that mNT5E protein was intact.

**Figure 2:**
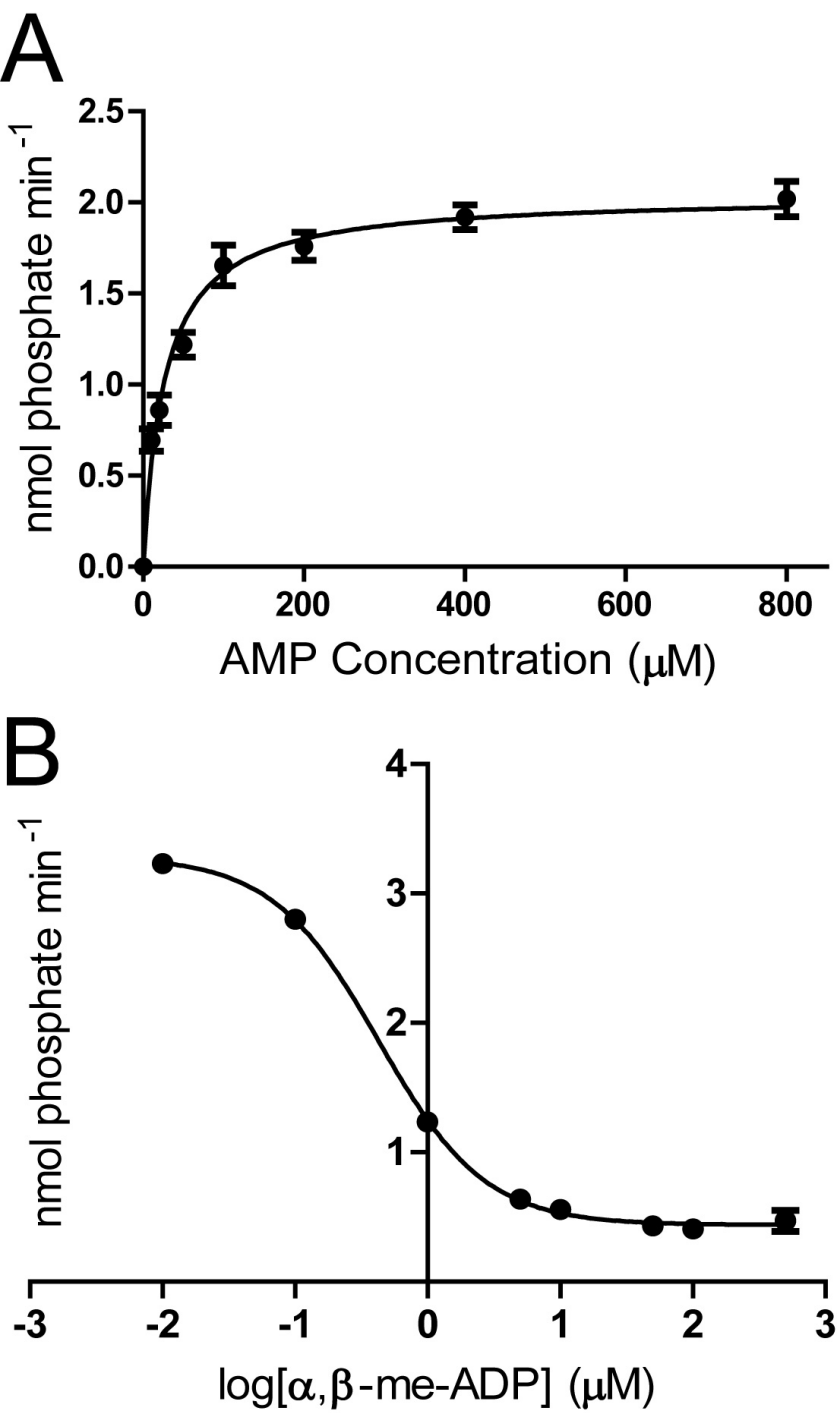
**mNT5E dephosphorylates AMP and can be inhibited by α,β-me-ADP**. (A) Plot of initial velocity at the indicated concentrations of AMP at pH 7.0. Reactions (n = 3 per point) were stopped after 3 min. (B) The indicated concentrations of α,β-me-ADP were added to reactions (n = 3 per concentration) containing mNT5E (0.07 μg/μL) and 400 μM AMP at pH 7.0. (A, B) Inorganic phosphate was measured using malachite green. All data are presented as means ± s.e.m. Some error bars are obscured due to their small size. GraphPad Prism 5.0 was used to generate curves.

The activity of secreted mNT5E was initially determined *in vitro*. Purified recombinant mNT5E protein dephosphorylated AMP with a *K*_*M *_of 26 μM (Figure 2A). This *K*_*M *_value is in agreement with the previously reported range of 1-50 μM using AMP as substrate at pH 7.0 [8,34]. Recombinant mNT5E was also inhibited by α,β-me-ADP (IC_50 _= 0.43 μM; Figure 2B), a commonly used inhibitor of NT5E [1]. Although recombinant NT5E was not used, others obtained a slightly higher IC_50 _value of 3.6 μM with this inhibitor [37]. For comparison, we found that α,β-me-ADP (0.01-500 μM) did not inhibit recombinant mouse PAP when AMP was used as substrate (data not shown). Production of recombinant mouse PAP was described previously [11].

### mNT5E has long lasting antinociceptive effects that are A_1_R dependent

**Figure 3:**
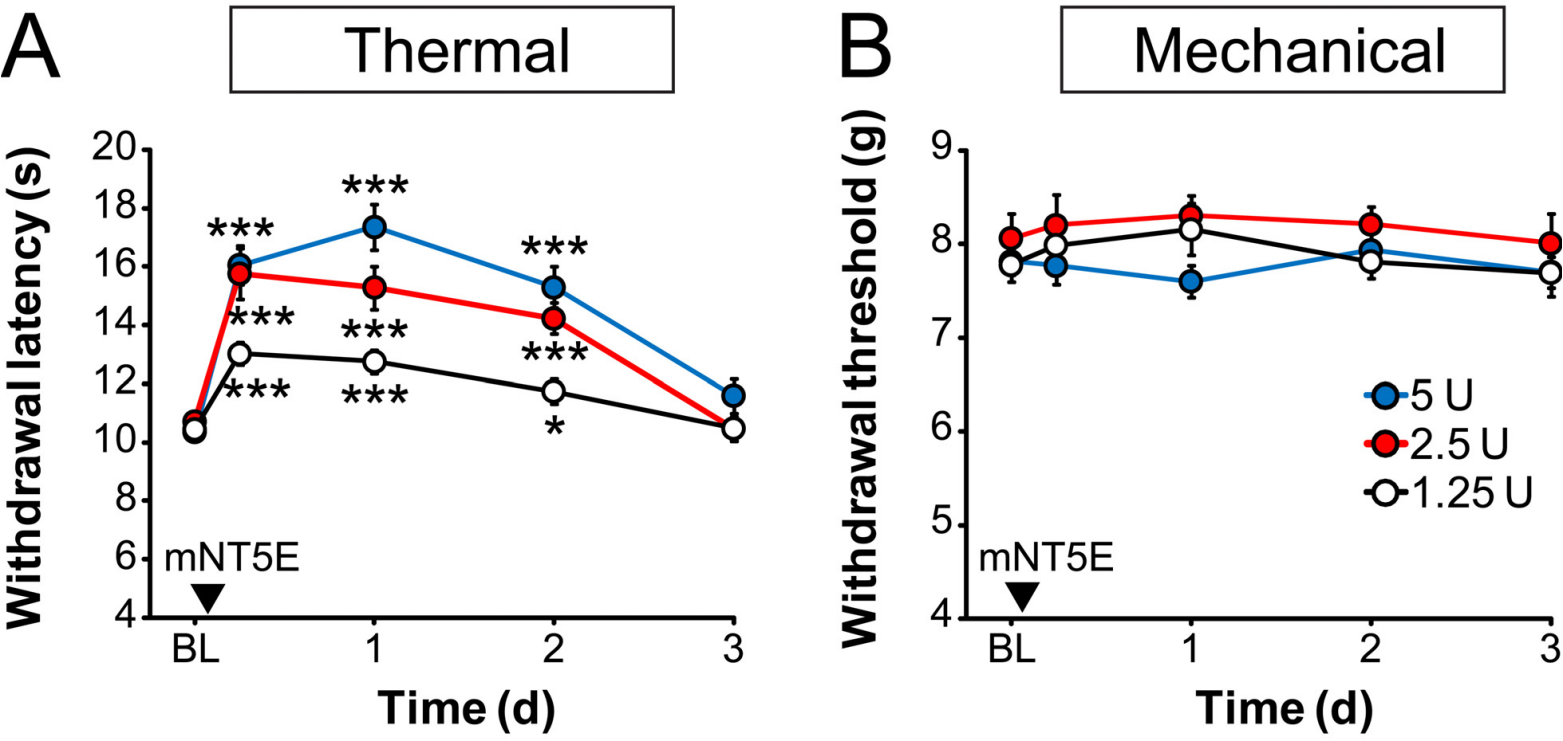
**Dose-dependent antinociceptive effects of intrathecal mNT5E**. Effects of the indicated amounts of mNT5E on (A) paw withdrawal latency to a radiant heat source and (B) paw withdrawal threshold (electronic von Frey apparatus). BL = Baseline. Injection (i.t.) volume was 5 μL. n = 10 wild-type mice were used per dose. Paired t tests were used to compare responses between BL values and later time points for each group. *P < 0.05, ** *P *< 0.005; *** *P *< 0.0005. All data are presented as means ± s.e.m.

We previously found that a single intrathecal injection of PAP had antinociceptive, antihyperalgesic and antiallodynic effects that lasted for three days and that were dependent on A_1_R activation [10,11]. To empirically identify an effective dose of mNT5E for *in vivo *studies and to determine if mNT5E had long lasting antinociceptive effects, we intrathecally injected wild-type mice with increasing doses of recombinant mNT5E protein (Figure 3). Time points were based on our previous studies with PAP [10,11]. We then measured noxious thermal and mechanical sensitivity before (baseline, BL) and after mNT5E injection. Six hours post i.t. injection, paw withdrawal latency to the noxious thermal stimulus was significantly increased relative to controls and remained elevated for two days at all doses tested (Figure 3A). Intrathecal injection of mNT5E did not alter mechanical sensitivity (Figure 3B) nor did it cause paralysis or sedation at any of the doses tested. We previously found that PAP (from human, cow and mouse) also had selective thermal but not mechanical antinociceptive effects in naïve mice and had no obvious motor side effects [10,11].

**Figure 4:**
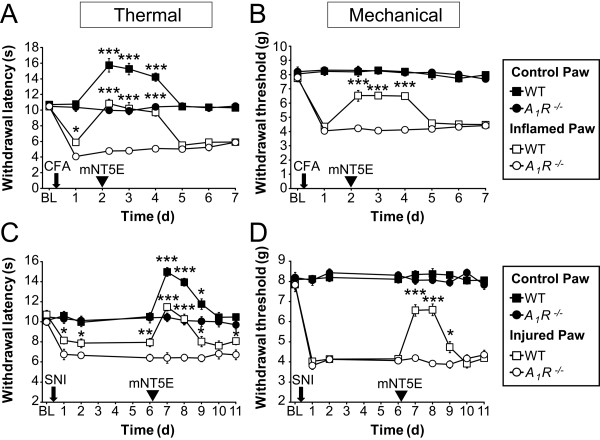
**mNT5E has antihyperalgesic and antiallodynic effects in WT mice following inflammation and nerve injury**. WT and *A*_1_*R*^-/- ^mice were tested for (A, C) noxious thermal and (B, D) mechanical sensitivity before (baseline, BL) and after injection of CFA into one hindpaw (A, B; arrow) or following nerve injury (C, D; SNI, arrow). (A, B) One or (C, D) six days later, mNT5E protein (1.7 U) was injected i.t. into all mice (arrowhead) then thermal and mechanical sensitivity was measured for several days. Inflamed/injured and non-inflamed/non-injured (control) hindpaws were tested. We used a 1.7 U dose to conserve protein and because it was nearly as effective as the 2.5 U dose (compare thermal antinociceptive effects in Figure 3A to panels A and C-control paws). Paired t testes were used to compare responses at each time point between genotypes (n = 10 animals per genotype). *P < 0.05, **P < 0.005, ***P < 0.0005. All data are presented as means ± s.e.m.

We next evaluated the antinociceptive effects of mNT5E in the CFA model of inflammatory pain and the SNI model of neuropathic pain. We used wild-type (WT) and *A*_1_*R*^-/- ^mice, to evaluate dependence on A_1_R activation. We used the contralateral (non-inflamed/non-injured) paw as a control. As seen previously [9,10,12], *A*_1_*R*^-/- ^mice displayed enhanced thermal hyperalgesia after CFA injection and after nerve injury relative to WT mice (Figure 4A, C). In both chronic pain models, a single i.t. injection of mNT5E had long lasting (at least 2 days) thermal antihyperalgesic and mechanical antiallodynic effects in the inflamed/injured paw of WT mice but not *A*_1_*R*^-/- ^mice (Figure 4A-D). Indeed, thermal sensitivity transiently returned to baseline levels in the injured/inflamed paws following mNT5E injection whereas mechanical sensitivity approached but did not reach baseline levels. Consistent with our dose-response study above, mNT5E had thermal but not mechanical antinociceptive effects in the control (non-inflamed/non-injured) paws of WT mice. mNT5E had no antinociceptive effects in *A*_1_*R*^-/- ^mice, highlighting a critical dependence on A_1_R activation. We previously found that control injections had no effect on thermal or mechanical sensitivity in these mouse models [10,11]. When combined with numerous studies of NT5E by others (reviewed by [2]), our data suggest that all the antinociceptive effects of mNT5E are due to production of adenosine and activation of A_1_R on DRG neurons and/or spinal neurons [38,39].

## Discussion

Adenosine and selective A_1_R agonists have well-studied antinociceptive effects in rodents and humans [26,40,41]. These agonists are often delivered intrathecally to avoid side effects associated with peripheral A_1_R activation [42]. However, A_1_R agonists do have side effects when delivered intrathecally at high doses, including overt motor paralysis [10,43]. These motor side effects are likely due to activation of A_1_R on motor neurons [39]. Our present study indicates that NT5E, an ectonucleotidase that hydrolyzes AMP to adenosine, provides an alternative means of activating A_1_R for therapeutic purposes without causing overt motor paralysis. Although PAP also has A_1_R-dependent antinociceptive effects when injected intrathecally [10,11], PAP shares no sequence similarity to NT5E. This lack of similarity made it impossible for us to determine *a priori *if recombinant mNT5E would be catalytically active, stable and effective when tested in live animals.

As previously discussed [11], ectonucleotidases are catalytically restricted in that they generate adenosine in proportion to substrate availability. Catalytic restriction limits the amount of adenosine produced and could explain why saturating doses of PAP or NT5E protein do not paralyze mice. Moreover, adenosine can be eliminated from the extracellular space by nucleoside transporters and metabolic enzymes, including adenosine deaminase and adenosine kinase [40,44]. These competing processes of adenosine production and elimination place an upper limit on how much extracellular adenosine is available to activate A_1_R in animals. In support of this, adenosine deaminase inhibitors and adenosine kinase inhibitors prolong the extracellular bioavailability of adenosine and have antinociceptive effects in animals [17,21,45,46].

Unexpectedly, our studies revealed that two molecularly distinct ectonucleotidases (PAP and NT5E) have pronounced antinociceptive effects that persist for an extended time period (2-3 days). This long duration of action *in vivo *could not have been predicted from the biochemical properties of these enzymes alone. More importantly, our findings suggest ectonucleotidases represent a new class of antinociceptive drugs with potential therapeutic advantages over adenosine, A_1_R agonists and inhibitors of adenosine metabolism. For example, the long duration of action of ectonucleotidases could be useful in situations where there is a need to provide a sustained level of A_1_R activation. Moreover, recombinant NT5E could be used to further study the role of NT5E in pain mechanisms and to study many other physiological processes that are regulated by NT5E [2,9].

## Methods

### Molecular biology

The GST-mNT5E baculovirus expression plasmid was generated by PCR amplification of mouse NT5E (nt 131-1696 from GenBank accession # NM_011851.3) using Phusion polymerase and a full-length expression construct of mNT5E as template. Primer sequences contained EcoRI sites (in bold italics) to facilitate cloning into pAcSecG2T (BD Biosciences). N-terminal primer: 5'-cgc***gaattc***attgggagctcacgatcctgcacaca. C-terminal (His)_6 _tag primer: 5'-gcg***gaattc***ttaatgatgatgatgatgatggaacttgatccgcccttcaacg. These primers amplify a product that contains the catalytic domain of mNT5E fused to the (His)_6 _epitope tag but that lacks the signal peptide and GPI anchor sequence (located at Ser523). This mNT5E PCR product was subcloned in frame with an N-terminal GST fusion tag, with a single thrombin cleavage site between the GST tag and the coding sequence of mNT5E. The final plasmid was sequence verified.

### mNT5E protein purification

The GST-mNT5E plasmid was used to generate recombinant mNT5E protein using the BD BaculoGold Expression System (BD Biosciences). Briefly, we infected Hi5 insect cells with high-titer recombinant baculovirus, incubated the cells for 48 hours at 27°C and then removed the cells from the supernatant by centrifugation. The supernatant containing secreted GST-mNT5E was filtered (0.45 μm pore size, Millipore) and concentrated in PBS (10 mM sodium phosphate, 140 mM NaCl, pH 7.4) using a Millipore cartridge with a 10 k retention cutoff. The concentrated supernatant was loaded onto a 5 mL GSTrap FF column (GE Healthcare) using a peristaltic pump at 4°C. Loading was performed overnight at a slow flow rate (0.4 mL/min. for 14-16 hours) to optimize binding of the GST-tagged protein. The column was then washed with 50 mL PBS. Purified thrombin (GE Healthcare, Cat. # 27-0846-01) was added to 2 mL of PBS (250 U thrombin/L of expression culture) and loaded onto the GSTrap column using a syringe. The on-column cleavage reaction proceeded for 16 hours at room temperature. The pre-loaded GSTrap column was then attached to an ÄKTA Explorer chromatography system with UV monitoring. Cleaved mNT5E and thrombin were eluted in PBS while the GST tag remained bound to the column. Fractions were monitored by SDS-PAGE to estimate purity, mNT5E concentration and cleavage efficiency (~80%). The cleaved mNT5E was separated from thrombin using a Superdex75 10/300 GL column attached to the ÄKTA Explorer system. Proteins were eluted in PBS at a flow rate of 0.5 mL/min. A maximum of 500 μL was injected per run. Fractions containing cleaved mNT5E were pooled, concentrated and then dialyzed against 0.9% saline. Protein purity was confirmed by SDS-PAGE, staining for total protein with GelCode Blue (Pierce/Thermo Scientific, Cat. # 24590) and western blotting with anti-NT5E antibody (R&D Systems, AF4488). Amersham full-range rainbow molecular weight markers (GE Healthcare) were used for SDS-PAGE and western blots. Recombinant mNT5E was kept at 4°C for short-term (1-2 months) use and at -80°C for long term storage.

### Enzyme assays

Enzymatic reactions (50 μL final) were carried out with recombinant mNT5E at 37°C for 3 minutes in 100 mM HEPES, pH 7.0, 4 mM MgCl_2 _with adenosine 5'-monophosphate disodium salt (Fluka, 01930) as substrate. Reactions were terminated by adding 950 μL of the malachite green color reagent [0.03% (w/v) malachite green oxalate, 0.2% (w/v) sodium molybdate, 0.05% (v/v) Triton X-100, dissolved in 0.7 M HCl] followed by incubation at room temperature for 30 minutes. Inorganic phosphate was quantified by measuring OD_650 _and comparing to an inorganic phosphate (KH_2_PO_4_) standard curve [47]. Unit (U) definition: 1 U hydrolyzes 1 nmol of AMP per minute at 37°C at pH 7.0. α,β-me-ADP was purchased from Sigma (M3763).

### Behavior

All behavioral experiments involving vertebrate animals were approved by the Institutional Animal Care and Use Committee at the University of North Carolina at Chapel Hill. C57BL/6 mice, 2-4 months old, were purchased from Jackson Laboratories. *A*_1_*R*^-/- ^mice were backcrossed to C57BL/6J mice for 12 generations [48,49]. Male mice were used for all behavioral studies and were acclimated to the testing room, equipment and experimenter for at least three days before testing. To further reduce variability in behavioral studies, mice were almost exclusively tested when in the resting or light sleep behavioral state [50]. The experimenter was blind to genotype during behavioral testing.

Thermal sensitivity was measured by heating one hindpaw with a Plantar Test apparatus (IITC) following the Hargreaves method [51]. The radiant heat source intensity was calibrated so that a paw withdrawal reflex was evoked in ~10 s., on average, in wild-type C57BL/6 mice. Cutoff time was 20 s. One measurement was taken from each paw per time point to determine paw withdrawal latency. Mechanical sensitivity was measured using a semi-flexible tip attached to an Electronic von Frey apparatus (IITC) as described elsewhere [52,53]. The force values obtained with this apparatus are higher than the force values obtained using calibrated von Frey filaments [53]. Three measurements were taken from each paw then averaged to determine paw withdrawal threshold in grams. To induce inflammatory pain, 20 μL complete Freund's adjuvant (MP Biomedicals) was injected into one hindpaw, centrally beneath glabrous skin, with a 30G needle. We performed spared nerve injury surgeries as described by Shields and colleagues [54]. mNT5E protein was diluted in 0.9% saline for intrathecal injection (5 μL/mouse) using the direct lumbar puncture method [55]. None of the mNT5E-injected mice displayed reduced mobility or paralysis following injection, as assessed by visually observing motor activity following injections.

## Competing interests

The authors declare that they have no competing interests.

## Authors' contributions

NAS carried out the behavioral studies and drafted figure legends, MKV carried out molecular and biochemical studies and drafted portions of the manuscript, MJZ designed the mNT5E expression construct, conceived of and designed the study and wrote the manuscript. All authors read and approved the final manuscript.

## References

[B1] Zimmermann H 5'-Nucleotidase: molecular structure and functional aspects Biochem J 1992 285 Pt 2 345 365 1637327 10.1042/bj2850345 PMC1132794

[B2] Colgan SP Eltzschig HK Eckle T Thompson LF Physiological roles for ecto-5'-nucleotidase (CD73) Purinergic Signal 2006 2 2 351 360 10.1007/s11302-005-5302-5 18404475 PMC2254482

[B3] Strohmeier GR Lencer WI Patapoff TW Thompson LF Carlson SL Moe SJ Carnes DK Mrsny RJ Madara JL Surface expression, polarization, and functional significance of CD73 in human intestinal epithelia J Clin Invest 1997 99 11 2588 2601 10.1172/JCI119447 9169488 PMC508104

[B4] Thompson LF Eltzschig HK Ibla JC Wiele CJ Van De Resta R Morote-Garcia JC Colgan SP Crucial role for ecto-5'-nucleotidase (CD73) in vascular leakage during hypoxia J Exp Med 2004 200 11 1395 1405 10.1084/jem.20040915 15583013 PMC1237012

[B5] Eltzschig HK Ibla JC Furuta GT Leonard MO Jacobson KA Enjyoji K Robson SC Colgan SP Coordinated adenine nucleotide phosphohydrolysis and nucleoside signaling in posthypoxic endothelium: role of ectonucleotidases and adenosine A2B receptors J Exp Med 2003 198 5 783 796 10.1084/jem.20030891 12939345 PMC2194189

[B6] Castrop H Huang Y Hashimoto S Mizel D Hansen P Theilig F Bachmann S Deng C Briggs J Schnermann J Impairment of tubuloglomerular feedback regulation of GFR in ecto-5'-nucleotidase/CD73-deficient mice J Clin Invest 2004 114 5 634 642 15343381 10.1172/JCI21851 PMC514589

[B7] Koszalka P Ozuyaman B Huo Y Zernecke A Flogel U Braun N Buchheiser A Decking UK Smith ML Sevigny J Targeted disruption of cd73/ecto-5'-nucleotidase alters thromboregulation and augments vascular inflammatory response Circ Res 2004 95 8 814 821 10.1161/01.RES.0000144796.82787.6f 15358667

[B8] Hunsucker SA Mitchell BS Spychala J The 5'-nucleotidases as regulators of nucleotide and drug metabolism Pharmacol Ther 2005 107 1 1 30 10.1016/j.pharmthera.2005.01.003 15963349

[B9] Sowa NA Taylor-Blake B Zylka MJ Ecto-5'-Nucleotidase (CD73) Inhibits Nociception by Hydrolyzing AMP to Adenosine in Nociceptive Circuits J Neurosci 2010 30 6 2235 2244 10.1523/JNEUROSCI.5324-09.2010 20147550 PMC2826808

[B10] Zylka MJ Sowa NA Taylor-Blake B Twomey MA Herrala A Voikar V Vihko P Prostatic acid phosphatase is an ectonucleotidase and suppresses pain by generating adenosine Neuron 2008 60 1 111 122 10.1016/j.neuron.2008.08.024 18940592 PMC2629077

[B11] Sowa NA Vadakkan KI Zylka MJ Recombinant mouse PAP Has pH-dependent ectonucleotidase activity and acts through A(1)-adenosine receptors to mediate antinociception PLoS ONE 2009 4 1 e4248 10.1371/journal.pone.0004248 19158948 PMC2617779

[B12] Wu WP Hao JX Halldner L Lovdahl C DeLander GE Wiesenfeld-Hallin Z Fredholm BB Xu XJ Increased nociceptive response in mice lacking the adenosine A1 receptor Pain 2005 113 3 395 404 10.1016/j.pain.2004.11.020 15661449

[B13] Gomes JA Li X Pan HL Eisenach JC Intrathecal adenosine interacts with a spinal noradrenergic system to produce antinociception in nerve-injured rats Anesthesiology 1999 91 4 1072 1079 10.1097/00000542-199910000-00028 10519511

[B14] Lavand'homme PM Eisenach JC Exogenous and endogenous adenosine enhance the spinal antiallodynic effects of morphine in a rat model of neuropathic pain Pain 1999 80 1-2 31 36 10.1016/S0304-3959(98)00193-6 10204715

[B15] Maione S de Novellis V Cappellacci L Palazzo E Vita D Luongo L Stella L Franchetti P Marabese I Rossi F The antinociceptive effect of 2-chloro-2'-C-methyl-N6-cyclopentyladenosine (2'-Me-CCPA), a highly selective adenosine A1 receptor agonist, in the rat Pain 2007 131 3 281 292 10.1016/j.pain.2007.01.013 17317007

[B16] Lee YW Yaksh TL Pharmacology of the spinal adenosine receptor which mediates the antiallodynic action of intrathecal adenosine agonists J Pharmacol Exp Ther 1996 277 3 1642 1648 8667233

[B17] Poon A Sawynok J Antinociception by adenosine analogs and inhibitors of adenosine metabolism in an inflammatory thermal hyperalgesia model in the rat Pain 1998 74 2-3 235 245 10.1016/S0304-3959(97)00186-3 9520238

[B18] Cui JG Sollevi A Linderoth B Meyerson BA Adenosine receptor activation suppresses tactile hypersensitivity and potentiates spinal cord stimulation in mononeuropathic rats Neurosci Lett 1997 223 3 173 176 10.1016/S0304-3940(97)13435-8 9080460

[B19] Holmgren M Hednar T Nordberg G Mellstrand T Antinociceptive effects in the rat of an adenosine analogue, N6-phenylisopropyladenosine J Pharm Pharmacol 1983 35 10 679 680 6139442 10.1111/j.2042-7158.1983.tb02867.x

[B20] Yoon MH Bae HB Choi JI Antinociception of intrathecal adenosine receptor subtype agonists in rat formalin test Anesth Analg 2005 101 5 1417 1421 10.1213/01.ANE.0000180994.10087.6F 16244004

[B21] Jarvis MF Mikusa J Chu KL Wismer CT Honore P Kowaluk EA McGaraughty S Comparison of the ability of adenosine kinase inhibitors and adenosine receptor agonists to attenuate thermal hyperalgesia and reduce motor performance in rats Pharmacol Biochem Behav 2002 73 3 573 581 10.1016/S0091-3057(02)00840-7 12151032

[B22] Aley KO Levine JD Multiple receptors involved in peripheral alpha 2, mu, and A1 antinociception, tolerance, and withdrawal J Neurosci 1997 17 2 735 744 8987795 10.1523/JNEUROSCI.17-02-00735.1997 PMC6573239

[B23] Zahn PK Straub H Wenk M Pogatzki-Zahn EM Adenosine A1 but not A2a receptor agonist reduces hyperalgesia caused by a surgical incision in rats: a pertussis toxin-sensitive G protein-dependent process Anesthesiology 2007 107 5 797 806 10.1097/01.anes.0000286982.36342.3f 18073555

[B24] Belfrage M Segerdahl M Arner S Sollevi A The safety and efficacy of intrathecal adenosine in patients with chronic neuropathic pain Anesth Analg 1999 89 1 136 142 10.1097/00000539-199907000-00023 10389791

[B25] Eisenach JC Hood DD Curry R Preliminary efficacy assessment of intrathecal injection of an American formulation of adenosine in humans Anesthesiology 2002 96 1 29 34 10.1097/00000542-200201000-00011 11752998

[B26] Eisenach JC Rauck RL Curry R Intrathecal, but not intravenous adenosine reduces allodynia in patients with neuropathic pain Pain 2003 105 1-2 65 70 10.1016/S0304-3959(03)00158-1 14499421

[B27] Giffin NJ Kowacs F Libri V Williams P Goadsby PJ Kaube H Effect of the adenosine A1 receptor agonist GR79236 on trigeminal nociception with blink reflex recordings in healthy human subjects Cephalalgia 2003 23 4 287 292 10.1046/j.1468-2982.2003.00511.x 12716347

[B28] Fukunaga AF Alexander GE Stark CW Characterization of the analgesic actions of adenosine: comparison of adenosine and remifentanil infusions in patients undergoing major surgical procedures Pain 2003 101 1-2 129 138 10.1016/S0304-3959(02)00321-4 12507707

[B29] Sollevi A Belfrage M Lundeberg T Segerdahl M Hansson P Systemic adenosine infusion: a new treatment modality to alleviate neuropathic pain Pain 1995 61 1 155 158 10.1016/0304-3959(94)00187-J 7644239

[B30] Karlsten R Gordh T Jr An A1-selective adenosine agonist abolishes allodynia elicited by vibration and touch after intrathecal injection Anesth Analg 1995 80 4 844 847 10.1097/00000539-199504000-00037 7893048

[B31] Lynch ME Clark AJ Sawynok J Intravenous adenosine alleviates neuropathic pain: a double blind placebo controlled crossover trial using an enriched enrolment design Pain 2003 103 1-2 111 117 10.1016/S0304-3959(02)00419-0 12749965

[B32] Eckle T Fullbier L Wehrmann M Khoury J Mittelbronn M Ibla J Rosenberger P Eltzschig HK Identification of ectonucleotidases CD39 and CD73 in innate protection during acute lung injury J Immunol 2007 178 12 8127 8137 17548651 10.4049/jimmunol.178.12.8127

[B33] Hart ML Much C Gorzolla IC Schittenhelm J Kloor D Stahl GL Eltzschig HK Extracellular adenosine production by ecto-5'-nucleotidase protects during murine hepatic ischemic preconditioning Gastroenterology 2008 135 5 1739 1750 e1733 10.1053/j.gastro.2008.07.064 18804111

[B34] Servos J Reilander H Zimmermann H Catalytically active soluble ecto-5'-nucleotidase purified after heterologous expression as a tool for drug screening Drug Dev Res 1998 45 269 276 10.1002/(SICI)1098-2299(199811/12)45:3/4<269::AID-DDR25>3.0.CO;2-B

[B35] Ogata S Hayashi Y Misumi Y Ikehara Y Membrane-anchoring domain of rat liver 5'-nucleotidase: identification of the COOH-terminal serine-523 covalently attached with a glycolipid Biochemistry 1990 29 34 7923 7927 10.1021/bi00486a021 2148114

[B36] Eisenhaber B Bork P Eisenhaber F Prediction of potential GPI-modification sites in proprotein sequences J Mol Biol 1999 292 3 741 758 10.1006/jmbi.1999.3069 10497036

[B37] Crane JK Shulgina I Naeher TM Ecto-5'-nucleotidase and intestinal ion secretion by enteropathogenic Escherichia coli Purinergic Signal 2007 3 3 233 246 10.1007/s11302-007-9056-0 18404437 PMC2096642

[B38] Schulte G Robertson B Fredholm BB DeLander GE Shortland P Molander C Distribution of antinociceptive adenosine A1 receptors in the spinal cord dorsal horn, and relationship to primary afferents and neuronal subpopulations Neuroscience 2003 121 4 907 916 10.1016/S0306-4522(03)00480-9 14580941

[B39] Reppert SM Weaver DR Stehle JH Rivkees SA Molecular cloning and characterization of a rat A1-adenosine receptor that is widely expressed in brain and spinal cord Mol Endocrinol 1991 5 8 1037 1048 10.1210/mend-5-8-1037 1658635

[B40] Sawynok J Liu XJ Adenosine in the spinal cord and periphery: release and regulation of pain Prog Neurobiol 2003 69 5 313 340 10.1016/S0301-0082(03)00050-9 12787573

[B41] Hayashida M Fukuda K Fukunaga A Clinical application of adenosine and ATP for pain control J Anesth 2005 19 3 225 235 10.1007/s00540-005-0310-8 16032451

[B42] Jacobson KA Gao ZG Adenosine receptors as therapeutic targets Nat Rev Drug Discov 2006 5 3 247 264 10.1038/nrd1983 16518376 PMC3463109

[B43] Sawynok J Adenosine and ATP receptors Handb Exp Pharmacol 2007 177 309 328 full_text 17087128 10.1007/978-3-540-33823-9_11

[B44] Kowaluk EA Jarvis MF Therapeutic potential of adenosine kinase inhibitors Expert Opin Investig Drugs 2000 9 3 551 564 10.1517/13543784.9.3.551 11060695

[B45] Keil GJ DeLander GE Spinally-mediated antinociception is induced in mice by an adenosine kinase-, but not by an adenosine deaminase-, inhibitor Life Sci 1992 51 19 PL171 176 10.1016/0024-3205(92)90566-8 1435056

[B46] Poon A Sawynok J Antinociception by adenosine analogs and an adenosine kinase inhibitor: dependence on formalin concentration Eur J Pharmacol 1995 286 2 177 184 10.1016/0014-2999(95)00444-P 8605954

[B47] Lanzetta PA Alvarez LJ Reinach PS Candia OA An improved assay for nanomole amounts of inorganic phosphate Anal Biochem 1979 100 1 95 97 10.1016/0003-2697(79)90115-5 161695

[B48] Hua X Erikson CJ Chason KD Rosebrock CN Deshpande DA Penn RB Tilley SL Involvement of A1 adenosine receptors and neural pathways in adenosine-induced bronchoconstriction in mice Am J Physiol Lung Cell Mol Physiol 2007 293 1 L25 32 10.1152/ajplung.00058.2007 17468137

[B49] Johansson B Halldner L Dunwiddie TV Masino SA Poelchen W Gimenez-Llort L Escorihuela RM Fernandez-Teruel A Wiesenfeld-Hallin Z Xu XJ Hyperalgesia, anxiety, and decreased hypoxic neuroprotection in mice lacking the adenosine A1 receptor Proc Natl Acad Sci USA 2001 98 16 9407 9412 10.1073/pnas.161292398 11470917 PMC55434

[B50] Callahan BL Gil AS Levesque A Mogil JS Modulation of mechanical and thermal nociceptive sensitivity in the laboratory mouse by behavioral state J Pain 2008 9 2 174 184 10.1016/j.jpain.2007.10.011 18088557

[B51] Hargreaves K Dubner R Brown F Flores C Joris J A new and sensitive method for measuring thermal nociception in cutaneous hyperalgesia Pain 1988 32 1 77 88 10.1016/0304-3959(88)90026-7 3340425

[B52] Cunha TM Verri WA Jr Vivancos GG Moreira IF Reis S Parada CA Cunha FQ Ferreira SH An electronic pressure-meter nociception paw test for mice Braz J Med Biol Res 2004 37 3 401 407 10.1590/S0100-879X2004000300018 15060710

[B53] Inoue M Rashid MH Fujita R Contos JJ Chun J Ueda H Initiation of neuropathic pain requires lysophosphatidic acid receptor signaling Nat Med 2004 10 7 712 718 10.1038/nm1060 15195086

[B54] Shields SD Eckert WA Basbaum AI Spared nerve injury model of neuropathic pain in the mouse: a behavioral and anatomic analysis J Pain 2003 4 8 465 470 10.1067/S1526-5900(03)00781-8 14622667

[B55] Fairbanks CA Spinal delivery of analgesics in experimental models of pain and analgesia Adv Drug Deliv Rev 2003 55 8 1007 1041 10.1016/S0169-409X(03)00101-7 12935942

